# Clinical Epidemiology, Pathology, and Molecular Investigation of Lumpy Skin Disease Outbreaks in Bangladesh during 2020–2021 Indicate the Re-Emergence of an Old African Strain

**DOI:** 10.3390/v14112529

**Published:** 2022-11-15

**Authors:** Rokshana Parvin, Emdadul Haque Chowdhury, Md Taohidul Islam, Jahan Ara Begum, Mohammed Nooruzzaman, Anja Globig, Klaas Dietze, Bernd Hoffmann, Eeva Tuppurainen

**Affiliations:** 1 Department of Pathology, Faculty of Veterinary Science, Bangladesh Agricultural University, Mymensingh 2002, Bangladesh; 2 Population Medicine and AMR Laboratory, Department of Medicine, Faculty of Veterinary Science, Bangladesh Agricultural University, Mymensingh 2202, Bangladesh; 3 Institute of International Animal Health/One Health, Friedrich-Loeffler-Institute, 17489 Greifswald, Germany; 4 Institute of Diagnostic Virology, Friedrich-Loeffler-Institute, 17489 Greifswald, Germany

**Keywords:** lumpy skin disease, clinical features, pathology, immunohistochemistry, genome analysis, Bangladesh

## Abstract

Lumpy skin disease (LSD) emerged in Bangladesh in mid-2019, leading to great economic losses for cattle farmers. This study describes the recent occurrence of the LSDV in Bangladesh and examines the clinical manifestation of the disease in local cattle breeds, characteristic epidemiological features, and pathological findings in affected animals. In addition, a full-genome sequencing of two local LSDV isolates was carried out. A total of 565 animals from 88 households were investigated, and 165 samples (skin lesions, saliva, nasal discharge, feces, and milk) were collected for virus detection. Pathology and immunohistochemistry were performed on nodule biopsies. Fever, nodular skin lesions, and swelling of the joints were the most common clinical manifestations. Skin lesions had a higher concentration of viral DNA compared to other sample types and were therefore selected for virus isolation and characterization. Pathology of the LSD skin nodules comprised a granulomatous reaction in the dermis and hypodermis that extended to the surrounding tissues. Development of the skin lesions started with swelling of keratinocytes with cytoplasmic vacuolation, vasculitis, panniculitis, thrombosis, and infarction. Altogether, the LSDV produced transmural, hemorrhagic, necrotizing, proliferative and ulcerative dermatitis. The LSD viral antigen was detected occasionally in the macrophages, epithelial cells, and vascular smooth muscle cells. The complete genome sequence analysis revealed that the two Bangladeshi field strains (BD-V392.1 and BD-V395.1) were distinct from the contemporary field strains and were closely related to the ancestral African Neethling strain. The findings of this study will improve the diagnosis, monitoring, and control of LSD in Bangladesh.

## 1. Introduction

Lumpy skin disease (LSD) is a notifiable, transboundary, high-impact disease of cattle and Asian water buffaloes (*Bubalus bubalis*). The causative agent is the lumpy skin disease virus (LSDV), which is a member of the genus *Capripoxvirus* within the family *Poxviridae* [1]. The LSDV is a large, enveloped virus with a linear double-stranded DNA [2,3]. Characteristic nodular lesions on the skin and the mucous membranes, enlarged superficial lymph nodes, and high body temperature are regarded as key indicators of the disease [4,5]. Infection with the LSDV is associated with emaciation, reduced milk production, infertility, and in some cases early embryonic death. Infected cattle often develop edematous swelling in their limbs and exhibit lameness [6]. LSD is distinguished by its moderate morbidity (10–45%) and low mortality rates (1–5%) [7].

LSD used to be considered a cattle disease in sub-Saharan Africa [6,8,9] and caused only sporadic outbreaks in Egypt [10], Israel [11], or elsewhere in the Middle East [12]. However, the geographical distribution of LSD began to expand dramatically after 2012 and the disease spread to Syria, Iraq, and Turkey and affected large numbers of cattle [13]. From Turkey, the disease spread to the Balkans, Caucasus, and Asia [14]. In recent years, one Asian country after another has reported the presence of LSD; the pattern of spread appears to follow the movements and trade of live cattle [15]. Bangladesh reported the first LSD outbreak in July 2019 [16] and immediately after that, the neighboring country India reported one in August of the same year [17]. According to the World Organization for Animal Health (WOAH) WAHIS database, LSD is present in almost all countries of Central, South, and Southeast Asia [18]. The most recent countries that reported LSD were Indonesia (February 2022), Afghanistan (February 2022), and Pakistan (March 2022). In addition, the susceptibility of some wild ruminants has been demonstrated [19,20,21,22]. LSD is transmitted mechanically by hemophagous vectors [23,24,25] that modulate the short-distance spread of the virus [26]. Transmission of the LSDV may also occur via direct or indirect contact [27,28] or via intrauterine transmission [29].

LSD has substantial economic consequences for all stakeholders of the cattle-farming industry due to the decreases in milk and beef production and the fertility problems, death, or treatment of severely affected animals. The costs of control and eradication measures, vaccinations, and consequences of the cattle movement and trade restrictions add to the economic losses. Countries that export cattle skins and hides are impacted by the decreased commercial value of the permanently scarred skins of affected animals. 

Detection of LSD cases relies mainly on passive surveillance; namely, on the recognition of highly characteristic clinical signs by people handling their animals. Farmers’ lack of awareness of LSD may hamper early detection, thereby enhancing widescale spread and causing more economic damage. Molecular methods such as real-time or conventional PCR assays allow rapid laboratory confirmation of tentative field suspicions. Serological methods such as ELISA, serum/virus neutralization tests, or the immunoperoxidase monolayer assay (IPMA) are mainly used for surveillance or research purposes [30]. LSDV isolation is time-consuming and requires high-biosafety-level laboratory facilities but may be needed for sequencing purposes or storing different strains in specific biobank collections. 

In Bangladesh, the first outbreaks of LSD were reported in commercial and backyard holdings in the Chattogram district [31]. Further outbreaks were later reported throughout the country [32,33,34]. Currently, LSD is one of the most economically important emerging livestock diseases in Bangladesh due to its wide distribution and huge cattle population in the backyard or household settings, which add to the increasing poverty and decreasing food security [32]. The disease is reported with varying clinical histories, and there is still a knowledge gap on the epidemiology and clinical presentation of LSD. In addition, more virus isolates that affect indigenous cattle across the Asian region need to be characterized. Survival of the virus in the environment, excreted different body fluids, or the skin lesions and scabs dropped off the skin of infected animals have not been sufficiently investigated. A recent partial gene sequencing analysis revealed unique genetic features of the LSDV in Bangladesh compared to the contemporary strains of other Asian countries [35]. However, complete genome sequences are still lacking that would facilitate a full understanding of the genomic features of the virus. Therefore, the aim of this study was to characterize the clinical manifestation of LSD and the pathomorphology of skin lesions shown by indigenous cattle in Bangladesh and to investigate the genetic background of the circulating virus. The presence of the LSDV was investigated in blood, saliva, nasal discharge, fecal, and milk samples. A histopathological examination of the skin biopsies and a molecular characterization of the virus found in the skin lesions were performed.

## 2. Materials and Methods

### 2.1. Study Area and Population 

The study was carried out in the Trishal, Bhaluka, Tarakanda, and Sadar upazilas (subdistricts) of the Mymensingh district in northern Bangladesh where several LSD outbreaks were noticed by farmers and government veterinarians from April 2020 to December 2021. According to the records of the District Livestock Office, the total number of cattle in the Mymensingh district was approximately 1.5 million with an average morbidity caused by any cattle disease of 25% and mortality of 5% each year. The ratio of dairy and beef cattle breeds was 6:5, of which most were indigenous with a few Holstein Friesian crossbred animals. The LSD outbreaks in the area were common throughout the year except during the winter months (December and January). The disease was, however, more common during the wet (May–July) and summer (August–October) seasons when vectors were breeding at cattle holdings. The usual temperature in Bangladesh fluctuates from 27 °C to 37 °C during the summer and wet seasons. The environment is ideal for the reproduction and growth of blood-feeding arthropods such as mosquitoes, biting flies, ticks, and fleas. Small cattle ranches in the rural areas are often unhygienic, which accelerates the abundance of vectors. Small-scale farmers typically feed their cattle in common pastures, nearby open fields, or roadside grass, which provides cattle with opportunities to mix with other herds and thus modulate the transmission of the LSDV.

### 2.2. Sample Collection

In this study, a total of 88 households comprising 565 cattle (mostly indigenous with a few crossbred) in the Mymensingh district (Supplementary Figure S1) were clinically examined. Each household had 2–15 animals. In the studied holdings, at least one animal showed febrile disease with nodular skin lesions. During the study period, 165 samples were taken from ongoing LSD outbreaks. The sample matrix included 56 skin lesions (scabs or nodules), 56 whole-blood samples, 13 saliva and 10 nasal-discharge swabs, 12 fecal samples, and 18 milk samples. Whole-blood samples were collected in EDTA vacutainers from the jugular vein while the other samples (skin lesions, saliva, nasal discharges, and feces) were collected in sterile 15 mL Falcon tubes containing transport media (Minimum Essential Media + 1% pen-strep). The milk samples were collected by hand in 2 mL tubes from all teats. The samples were transported to the laboratory in cooling boxes and stored at −70 °C until tested. For the histopathology, four skin nodule biopsies were taken from affected animals from four subdistricts at different stages of disease development. They were collected in a plastic container containing 10% neutral buffered formalin. 

### 2.3. Clinical and Epidemiological Data Collection

To obtain a better understanding of the epidemiology of LSD in the Bangladesh settings, data were collected in collaboration with the local veterinary services using a standard, well-structured questionnaire. The basic demographic information included farmers’ socioeconomic situation, farm biosecurity in place, animal housing practices, clinical manifestation of LSD in animals and the length of the disease, cattle vaccination history, and contacts with other cattle of the same household or within the community. Descriptive statistics was used to calculate the proportions of clinical signs, and a box-and-whisker plot was drawn for the quantitative variables such as the temperature, duration of illness, and age of skin lesions.

### 2.4. Quantitative PCR

Total DNA was extracted from skin lesions (scabs or nodules), blood, saliva, nasal swabs, fecal, and milk samples using the DNeasy Blood & Tissue Kit (Qiagen, Hilden, Germany). DNA was then tested using a capripox generic real-time PCR assay using a previously described primer and probe mix [36,37] that amplified a part of the P32 envelope protein gene. The qPCR reaction was prepared in a total volume of 12.5 µL consisting of 6 µL of Luna^®^ Universal Probe qPCR Master Mix (NEB, Hitchin, UK), 2 µL of primer–probe mix, 2 µL of nuclease-free water, and 2.5 µL of template DNA. The PCR was carried out on an ABI 7500 Fast thermal cycler (Applied Biosystems, Waltham, MA, USA) with a cycle condition of one cycle at 95 °C for 2 min (activation) followed by 40 cycles at 95 °C for 15 s (denaturation) and 60 °C for 30 s (annealing and extension). The cycle threshold (Ct) values obtained at ≤35 from the clinical samples were considered positive.

### 2.5. Virus Isolation 

Selected PCR positive samples with the Ct values between 18 and 25 were used for the virus isolation and sequencing. Confluent monolayers of Madin–Darby bovine kidney (MDBK) cells were utilized for LSDV isolation. The infected cells were monitored daily for 7 days for a cytopathic effect (CPE). The virus was harvested and identified using qPCR as described above. 

### 2.6. Sequencing and Phylogeny 

Two LSDV isolates—LSDV/Mymensingh-4_V392.1/Bangladesh/2021 (BD-V392.1) and LSDV/Mymensingh-7_V395.1/Bangladesh/2021 (BD-V395.1)—were selected for the whole-genome sequencing using the Illumina technology. The selection of samples for virus isolation and sequencing was based on the previous knowledge that the highest viral loads are found in skin lesions. Therefore, the skin samples for sequencing were collected from two independent outbreaks from the animals showing most severe clinical signs with multiple skin lesions. Genomic DNA of cell-culture-propagated LSDV from Bangladesh was extracted using the MasterPure Complete DNA and RNA Purification Kit (Lucigen/Biozym Scientific GmbH, Hessisch Oldendorf, Germany) according to the manufacturer’s instructions to prepare the samples for sequencing on the Illumina HiSeq 2500 (Illumina, San Diego, CA, USA) platform. Subsequently, a sample was shipped to the ISO 17025-accredited Eurofins Genomics lab (Eurofins Genomics Germany GmbH, Ebersberg, Germany) for high-throughput Illumina sequencing (Illumina). The sample was prepared for sequencing following the company’s workflow. In total, more than 10 million paired reads were produced for further analyses of both LSDV strains. 

Consensus sequences were generated with the mapping tool implemented in Geneious v.11.1.5 (Biomatters, Auckland, New Zealand) using different LSDV reference sequences (NC_003027, MH893760, and KX683219). The respective full-genome sequence that was obtained was submitted to GenBank under the accession numbers LSDV/Mymensingh-4_V392.1/Bangladesh/2021 (BD-V392.1) OP688128 and LSDV/Mymensingh-7_V395.1/Bangladesh/2021 (BD-V395.1) OP688129.

The obtained complete sequences of two isolates (BD-V392.1 and BD-V395.1) along with other representative field and vaccine strains of LSDV, sheeppox virus, and goatpox virus were downloaded from a public domain site (NCBI GenBank). The selected sequences were then subjected to multiple alignment using the MAFFT software’s online version (https://mafft.cbrc.jp/alignment/server; accessed on 13 September 2022). The phylogenetic analysis was performed using the neighbor-joining method and Jukes–Cantor substitution model incorporated in the MAFFT package with the confidence intervals estimated by applying a 1000-sample bootstrap algorithm [38]. Finally, the phylogenetic trees were annotated and visualized using FigTree v1.4.2 (http://tree.bio.ed.ac.uk/software/figtree; accessed on 14 September 2022) and Inkscape 1.0 (https://inkscape.org; accessed on 14 September 2022). 

### 2.7. Gross and Histopathological Examination

Four skin nodules were collected at different stages of disease development that tested positive according to qPCR. Gross skin lesions were recorded prior to the histopathological examination. The specimens were fixed in 10% neutral buffered formalin for 7 days. After trimming and embedding in paraffin, 2–3 μm-thick sections were stained with hematoxylin and eosin (HE). In addition, Masson’s trichrome, a three-color staining procedure, was used as a selective stain for collagen fibers that stained blue; whereas muscles, cytoplasm, and keratin were stained red (https://microbenotes.com/massons-trichrome-staining/; accessed on 20 July 2022). Slides were scanned using a Hamamatsu S60 scanner (Hamamatsu Photonics, K.K., Shizuoka, Japan); the evaluation was performed using NDPview.2 plus software (Version 2.8.24, Hamamatsu Photonics, K.K. Japan). 

### 2.8. Immunohistochemistry (IHC)

Consecutive slides were processed for immunohistochemistry according to the standardized procedures of the avidin–biotin–peroxidase complex method. Briefly, 2–3 µm sections were mounted on adhesive glass slides, dewaxed in xylene, and rehydrated in descending graded alcohols. Endogenous peroxidase was quenched with 3% H_2_O_2_ in distilled water for 10 min at room temperature. Antigen heat retrieval was performed using 10 mM citrate buffer (pH 6.0) for 10 min in a microwave (700 watt) followed by a cooling period. Nonspecific antibody binding was blocked by goat normal serum (1:2 dilution in PBS) for 30 min at room temperature. The primary antibody against the LSD virus antigen was applied for one hour at room temperature (rabbit anti-LSD-144 polyclonal antibody at 1:200 dilution in PBS; obtained from IVD, FLI, Germany). As a negative control, consecutive sections were incubated with pre-immune rabbit serum. The secondary biotinylated goat anti-rabbit antibody was applied for 30 min at room temperature (Vector Laboratories, Burlingame, CA, USA; 1:200 dilution in PBS). A positive control slide (archived skin samples from experimental study) was included. Color was developed by incubating the slides with avidin–biotin–peroxidase complex (ABC) solution (Vectastain Elite ABC Kit; Vector Laboratories) followed by exposure to 3-amino-9-ethylcarbazole substrate (AEC, Dako, Carpinteria, CA, USA). The sections were counterstained with Mayer’s hematoxylin and coverslipped, the slides were scanned, and the evaluation was performed using NDPview.2 plus software. 

## 3. Results

### 3.1. Description of the Clinical Features

Among the 565 cattle from 88 households, 191 were clinically affected (33.8%) and 374 were apparently healthy. At the time of the investigation, the affected animals showed either ongoing clinical disease (n = 56) or had recovered (n = 135) from LSD within the last 6 months. Cows were most often affected (36.6%) followed by calves (27.2%), bulls (19.4%), and heifers (16.8%). 

**Figure 1:**
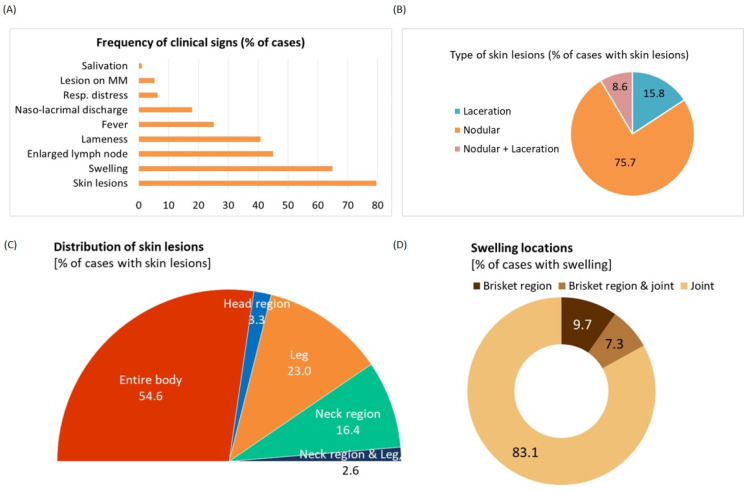
Clinical observations and distribution of lesions of the affected cattle population. (**A**) Frequency of clinical signs shown by the affected cattle. (**B**) Pie chart showing the type of skin lesions that were classified as nodular, laceration, and both. (**C**) Half pie chart showing the distribution of skin lesions in different parts of the body. (**D**) Pie chart revealing the occurrence of swelling in different body parts.

The most frequently observed clinical signs included skin lesions, swellings, enlargement of lymph nodes, lameness, and fever. Less frequent clinical signs included salivation, lesions on the mucous membranes, respiratory distress, and naso-lacrimal discharge, (Figure 1A). In 75.7% of the examined animals the skin lesions were at the nodular stage, indicating a recent infection. In 15.8% cases, sequestration was recorded, indicating a longstanding infection. Both nodular and sequestration lesions were recorded in 8.6% cases (Figure 1B). In a majority of cases, skin lesions were disseminated throughout the entire body (54.6%) or found to be restricted to particular sites such as the head (3.3%), only legs (23%), only neck (16.4%), or a combination of neck and legs (2.6%) (Figure 1C). Swollen joints (83.1%) were found mostly at the fetlock, hock, and/or carpal joints; edema was recorded in ventral tissues of the chest (brisket) and abdomen (Figure 1D). 

The body temperature of the infected cattle ranged from 102 to 106 °F (38.9 to 41 °C) but in most cases, it was between 102 and 103 °F (38.3 to 39.4 °C). We were able to estimate the tentative age of the skin lesions by subtracting the date of the sample collection or clinical examination from the date of the first appearance of the skin lesions observed by the farmers. The overall duration of illness was calculated from the onset of disease to complete recovery. Generally, the skin lesions lasted for 3 to 110 days with an average of 19.9 days, and the average duration of illness was 2.9 weeks with a range of 1 to 14 weeks. The summary of the clinical observations is shown in Supplementary Figure S2.

The appearance of multiple skin nodules of varying sizes (mostly of a 1–3 cm diameter) throughout the body was a common clinical feature. Some cattle showed skin ulcerations on mucous membranes, particularly in the muzzle, and on the limbs. In the examined cattle, the severity of the clinical manifestations varied from mild to severe. In some animals, the nodules densely covered the entire body. It was common to observe the top of the skin nodule to slough off, leaving a deep ulcer that often became infested with maggots in the advanced stage of the infection. In some cases, the sores were swiftly covered with scabs without oozing pus. Multiple nodules on the lower parts of the body and swelling of the joints were also evident. In the recovered animals, the nodules gradually disappeared, revealing normal-looking skin. An overview of the above clinical manifestations exhibited by the affected cattle are shown in Supplementary Figure S3.

### 3.2. Detection of LSDV

**Figure 2:**
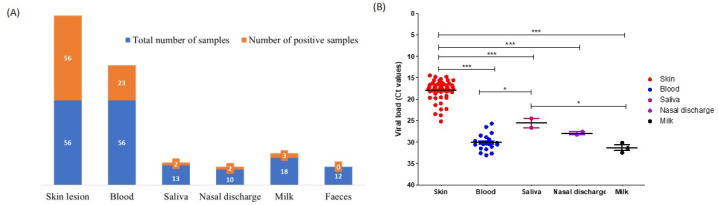
Detection of LSD viral genome with qPCR. (**A**) Bar chart showing the number of positive cases in various samples compared to the total number of samples collected. (**B**) Scatter chart describes the detection limit (calculated from the Ct values at qPCR) for different positive samples. The level of significance was calculated using a one-way ANOVA test (Tukey’s multiple comparison test). *** *p* ≤ 0.0001; * *p* ≤ 0.05.

To detect viral DNA, a previously established pan-capripoxvirus qPCR method was used to test the skin lesions and blood in the EDTA, nasal discharge and saliva swabs, feces, and milk samples collected from cattle with an ongoing infection (n = 56). The LSD viral DNA load was highest in the skin lesions: all skin lesion samples (56) tested positive (100%) with Ct values ranging between 14.5 and 25.2. LSDV DNA was less frequently detected in blood (41.07%), saliva (15.38%), nasal discharge (20%), and milk (16.66%) samples, whereas none of the fecal samples tested positive (Figure 2A). In addition, the Ct values in these positive samples varied between 25 and 35, which indicated a significantly lower viral load compared to those of the skin lesions (Figure 2B). 

### 3.3. Macroscopic and Microscopic Observations

**Figure 3:**
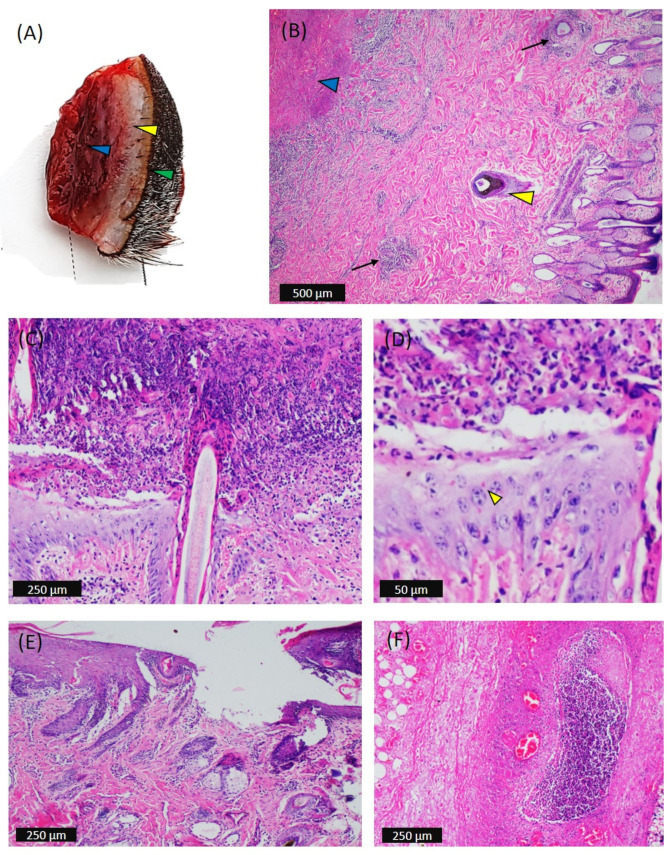
Gross and microscopic pathology of the skin nodules in affected cattle. (**A**) Biopsy of a skin nodule that was 2 cm in diameter that provided gritty sound when cut and showed hemorrhagic subcutis (blue arrowhead), pale dermis (yellow arrowhead), and haired epidermis (green arrowhead). (**B**) Epidermis: epidermal cytoplasmic swelling and mononuclear infiltrate. Dermis: diffuse proliferation of mononuclear cells between the dense irregular connective tissue and reticular layers and in the perivascular space (black arrow). Sweat and sebaceous glands were found dilated, and there was intracellular edema of sebaceous gland cells. The hair follicle matrix epithelia were found to be highly hyperplastic (yellow arrowhead). Subcutis: presence of an infarct (blue arrowhead). (**C**) Epidermis and dermis: vacuolation, swelling, and adhesion of scabs with proliferative stratum basale, intraepidermal necrosis, and accumulation of scale crust leaving ulcer underneath; in addition, the dermis was hemorrhagic, edematous, and infiltrated with mononuclear cells. (**D**) Occasionally, keratinocytes were consistent with intracytoplasmic inclusion bodies (yellow arrowhead). (**E**) Vacuolation and swelling of keratinocytes, epidermal proliferation of basal cells, infiltration of round histiocytic cells in the reticular layer of skin, swelling, and vacuolation of glandular epithelium and focal ulceration. (**F**) Subcutis: presence of focal aggression of mononuclear cells, congestion, necrosis, and lysis of subcutaneous fat cells.

In the biopsy of an unulcerated nodule that was 2 cm in diameter, three distinct zones were visible: a hemorrhagic red necrotic (infarct) zone at the center of the subcutis, a middle zone (dermis) that was whitish and provided a crepitating sound when cut, and the outer unulcerated or intact pigmented epidermis with hair follicles (Figure 3A). Histology of the nodule also confirmed the distinct zones (Figure 3B). Epidermal lesions started with vacuolar degeneration of keratinocytes and progressed to (micro)vesicle formation and partial erosion due to rupture of the vesicles (Figure 3B). Most of the keratinocytes appeared to be hypereosinophilic (degenerated); at the periphery of the lesion, keratinocytes were found with hyperchromatic basophilic nuclei (Figure 3B). At a later developmental stage of the nodules, most degenerated keratinocytes necrotized, adhered, accumulated, and formed a crust over the ulcer underneath (Figure 3C). Proliferation of the stratum basale of the surrounding area was detected, including a thickened epidermis with occasional intracytoplasmic inclusion bodies in the keratinocytes (Figure 3D). Epidermal proliferation of basal cells continued with infiltration of round histiocytic cells in the reticular layer of the skin; swelling and vacuolation of glandular epithelium and focal ulceration was evident (Figure 3E,F).

**Figure 4:**
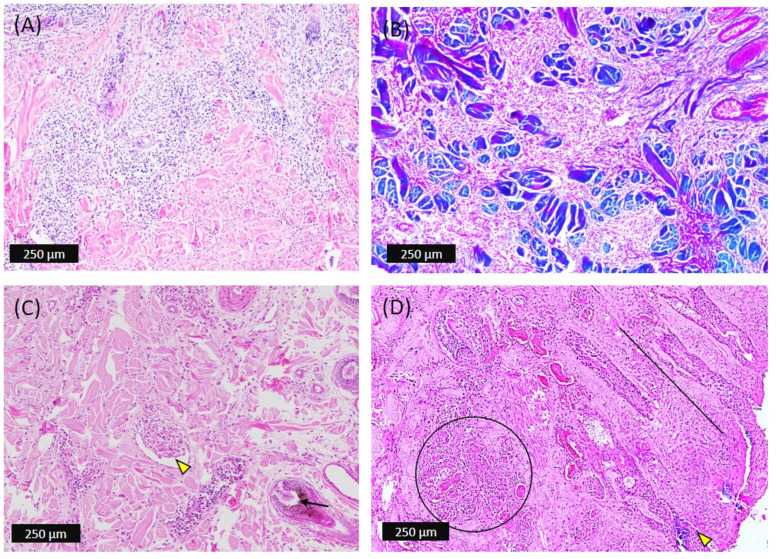
Microscopic pathology of the skin nodules developed in dermis of affected cattle. (**A**) Diffuse to nodular proliferation of mononuclear cells in the dense irregular connective tissue layer of dermis as well as centering the blood vessels. (**B**) Abundance of connective tissue fibers (blue) after using Masson’s trichrome stain. (**C**) The inflammatory cells adhered to lumen and around blood vessels (yellow arrowhead); the hair follicles’ epithelial cells are degenerated and hyperplastic (black arrow). (**D**) Dermis showing massive necrotizing dermatitis with multiple infarcts (black circle) and formation of excretory sinuses (black line) with invaded bacteria (yellow arrowhead).

The most striking lesions were observed in the dermis. The lesions started with infiltration of round histiocytic cells at the perivascular space; the later stages revealed multifocal diffuse and nodular infiltration of the histiocytic cells within the dense irregular connective tissue and reticular layers of the dermis (Figure 4A) that clearly showed as blue in the Masson’s trichrome stain (Figure 4B). In a few animals, the round inflammatory cells were aggregated and invaded the blood vessel wall (vasculitis). The hair follicles’ root sheath epithelial cells were degenerated and consistent with inclusion bodies; matrix epithelial cells of the follicle were found to be hyperplastic (Figure 4C). The blood vessels were congested, the sweat glands were dilated, and intracellular edema of the sebaceous gland epithelium was evident. Massive necrotizing dermatitis with multiple infarcts and formation of excretory sinuses with invaded bacteria were also evident at later stages of the disease (Figure 4D).

In the subcutis, the pannicular fat was severely affected and a hemorrhagic infarct was visible at the center of the skin nodule (Figure 3B). There was the presence of focal aggression or diffuse proliferation of mononuclear cells, congestion, necrosis, and lysis of subcutaneous fat cells (Figure 3F). The macrophages were found to be scatter-distributed in the necrotic subcutis and were consistent with the eosinophilic inclusion bodies (Figure 4C)

**Figure 5:**
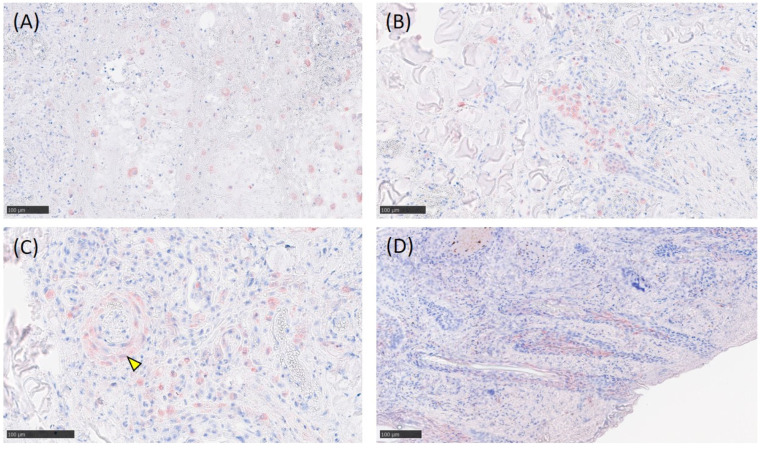
LSDV antigen in tissues. (**A**) Presence of LSD virus antigen in macrophages. (**B**) Hair follicle epithelial cells, endothelium, and smooth muscle cells. (**C**) Smooth muscle cells of the blood vessels (yellow arrowhead), histocytes, and macrophages. (**D**) In the hair follicle epithelium, the reaction was developed by using AEC and counterstained with hematoxylin.

Immunohistochemistry revealed LSDV antigen laden macrophages throughout the subcutis (Figure 5A). The LSDV antigen was detected occasionally in hair follicle epithelial cells, the endothelium and smooth muscle cells of the blood vessels, and histiocytic cells (Figure 5B–D).

### 3.4. Phylogenetic Relationships

**Figure 6:**
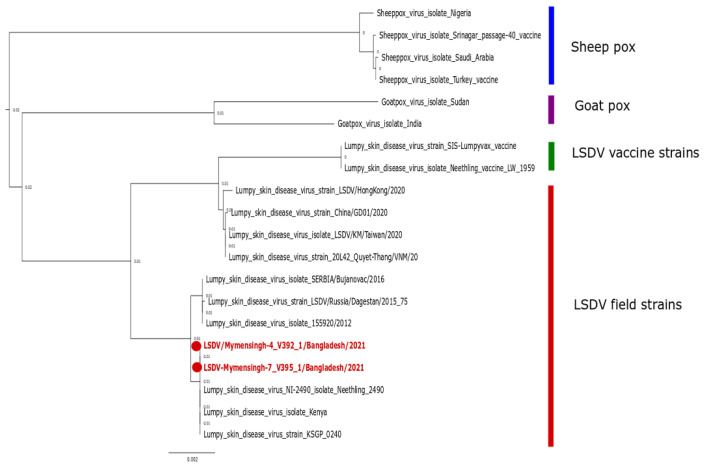
Phylogenetic analysis of Bangladeshi LSDV isolated from affected cattle. Neighbor-joining tree based on complete genome (150,773 bp) of capripoxviruses. LSDV from Bangladesh are highlighted by the red dots and taxon and clustered with older field strains of LSDV. Trees were generated after the selection of the best-fitted model using the MAFT software’s web version and viewed in Figtree V1.4.4.

Sequencing confirmed the presence of LSDV in the Bangladeshi small-holding cattle population. A phylogenetic analysis was performed to determine the relationship of the Bangladeshi LSDV with other contemporary LSDV field strains, vaccine strains, and other representative members of the capripoxviruses. The phylogenetic tree showed separate clusters for the LSDV field strain, vaccine strain, sheeppox virus, and goatpox virus (Figure 6). More intriguingly, both sequenced Bangladeshi field strains (LSDV/Mymensingh-4 V392.1/Bangladesh/2021 (BD-V392.1) and LSDV/Mymensingh-7 V395.1/Bangladesh/2021 (BD-V395.1)) were closely related to ancestral LSDV field strains collected from Africa before 1960 (Neethling 2490 or KSGP 0240). Furthermore, the BD-V392.1 and BD-V395.1 strain remained distinct from contemporary field strains from other Asian countries such as Hong Kong, China, Taiwan, and Vietnam.

## 4. Discussion

The current study investigated the LSD outbreaks during 2020–2021 in selected regions of Bangladesh. Clinical and cutaneous pathomorphological features were analyzed and LSDV was detected using real-time PCR in various sample materials. The viral antigen was disseminated in the epidermis, dermis, and subcutis of the skin nodules. Two Bangladeshi strains (BD-V392.1 and BD-V395.1) were completely sequenced, and a phylogenetic characterization was performed.

The first LSD outbreak in Bangladesh occurred in July 2019 and spread quickly over several districts [31]. Since then, the virus has been circulating among the cattle population of the entire country. Facultative vaccination with an attenuated goatpox vaccine locally produced by the Livestock Research Institute (LRI), Bangladesh, was performed by the government with only a limited coverage, leaving many small holdings unvaccinated. This study focused on the cattle farms where LSD outbreaks occurred without any LSDV vaccination history. 

The clinical observations of this study were in line with the published data from many other affected countries. Classical LSD symptoms such as anorexia, fever, enlargement of lymph nodes, appearance of skin nodules covering either the entire body or parts of the body, lameness, and swelling of the fetlock and hock joints were commonly detected [39,40,41]. Thus, the farmers and outbreak investigators easily learned to identify the typical clinical signs of LSD. The duration of illness and the total length of the persistence of skin nodules were almost similar, usually between two and four weeks. The morbidity amongst the study population of 33.8% was slightly higher than reported in previous studies [4,12,42] with two animals found dead; one calf died from severe respiratory distress, and one heifer died from a secondary maggot infestation in the LSD skin lesions. When examining at the epidemiological parameters in this study, clinically LSD occurrence was higher in calves (27.2%) and cows (36.6%) compared to heifers (16.8%) and bulls (19.4%). The first exposure to the LSD virus of young animals with an immature immune system or dairy cows experiencing high production stress may have increased their mortality rate [9]. 

The outbreaks were more common within a radius of 4–5 km from the infected holdings. This was probably related to the common practice of backyard farmers using communal pastures, which allows cattle to mix with other herds. On the other hand, there was no evidence of the development of clinical signs or skin nodules in all animals in infected holdings or epidemiological units, indicating that some of the unvaccinated cattle were immunologically protected or there was inefficient transmission between animals. Further seroprevalence studies may be required to explain the validity of this finding. 

The diagnosis of LSD is mainly based on the detection of highly characteristic clinical signs and molecular laboratory testing of samples from suspected animals. In this investigation, we performed a thorough histological and immunohistochemical examination of skin biopsies collected from LSD-affected animals. The virus caused transmural, hemorrhagic, necrotizing, proliferative, and ulcerative dermatitis in affected cattle. Multiple infarcts were detected in the dermis and subcutis of infected animals, similarly to the previous studies [43,44]. An infarct is usually developed due to occlusion or hindrance of the flow in blood vessels. In this study, we found proliferation of round histiocytic cells in the perivascular space of nearly damaged blood vessels. Similar findings for histiocytic cells were reported by Sanz-Bernardo and coworkers (2020) in their recent immunohistochemical study [44]. In addition, in some animals we found round inflammatory cells aggregated and invaded into the wall of the blood vessels (vasculitis with thrombus formation). This phenomenon might have blocked blood flow, leading to the development of an infarct in the affected skin. The changes associated with the blood vessels may indicate that viruses initially were distributed through the blood vessels and later spread through the infected cells. However, the cause of the associated blood vessel changes is unclear. El-Neweshy and coworkers (2013) identified the viral antigen in macrophages and in degenerated epithelial cells, not in the endothelial cells [43]. For this reason, they hypothesized an immune-mediated vascular damage that leads to infarction. In our study, the viral antigen was detected in the smooth muscle cells and endothelium of the blood vessels, histiocytic cells, and the macrophages of the dermis and subcutis. This may indicate that the virus was localized in the blood vessel wall/tissues, which in turn initiated the inflammation. However, the reason for histiocytic cell proliferation in LSDV infections requires further investigation. Histiocytes are a type of Langerhans cell with phagocytic and antigen-processing functions; these cells are abundant in the skin. Proliferation of these cells around blood vessels is apparently a defense against the antigen. In 2020, Sanz-Bernardo and colleagues were also able to immunohistochemically identify the antigen in histiocytic and fibroblastic cells of skin nodules [44]. The differences in antigen labeling in various studies, including ours, still need to be clarified regarding whether they were caused by problems in antigen unmasking of tissues or by the presence of very few viruses due to differences in clinical stages. However, quantifying the virus could provide further support for our finding that the viral load was higher on affected skin. Later in the infection, the nodular lesions seemed to have disappeared, which may have been due to the physiological loss in the histiocytic cells from the site. Masson’s trichrome staining showed that the nodules were rich in collagen, elastic fibers, and histiocytic cells but showed no signs of fibrous connective tissue proliferation. Usually, fibrous connective tissue persists in a progressive manner at the site of proliferation even if the animal recovers, which was not found with the LSDV. This histological feature correlated with clinical appearance of fully recovered cattle, as at some points no nodular lesions were seen on the skin. However, scarring of the epidermis occurred in recovered animals, particularly when the animals had ulcers and maggot infestations. The virus was usually detected in 5- to 25-day-old skin nodules. Scab formation was noticed at a more advanced stage, typically in 20–35-day-old skin nodules or even later after infection. The formation of intracytoplasmic inclusion bodies in skin lesions was another common feature observed in previous studies [45,46] and in the present study. Furthermore, vasculitis, congestion, edema, and necrosis were common in skin layers as described in other studies [43,44,47].

Although the clinical manifestation of LSD is straightforward, molecular detection via real-time PCR is most frequently used to confirm field cases and distinguish the LSDV field strain from the vaccine strain [48,49,50]. Different types of samples such as skin lesions, blood, nasal and saliva swabs, and milk have been used to detect LSD viral DNA [51,52]. In addition, in this study we collected skin nodules that were known to contain the highest viral loads for antigen detection. Other sample types such as blood, nasal and saliva swabs, and fecal and milk samples were found to be less effective for successful virus detection or may require a specific timing for sampling. However, noninvasive sampling methods have been shown to detect LSDV infections at the group or herd level with high sensitivity [36]. Milk samples, whether at the individual animal level or as pooled samples from bulk milk, are commonly used for screening purposes in dairy herds. In the context of LSD, they have shown to be more appropriate for the detection of antibodies rather than the viral genome [53]. All 56 skin samples collected from animals with an ongoing infection tested positive for LSDV (100%); however, testing of blood, milk, saliva, or nasal swabs from the same outbreak appeared to be slightly less sensitive. A higher percentage of positive skin samples was also found in previous studies [4,52,54]. Therefore, skin nodules and scabs remain the preferred sample types for LSDV detection. Virus isolation using Madin–Darby bovine kidney (MDBK) cells is another gold standard protocol for LSDV detection [55] that was also successfully used in the current study. 

Although the exact origin of the currently spreading strain of LSDV is not known with certainty, it is highly likely that LSDV entered Bangladesh either through vectors or through live cattle imported legally or illegally from neighboring countries. In this study, the whole-genome sequences of two Bangladeshi strains of LSDV obtained from two independent field outbreaks shed further light on the molecular epidemiology of the virus. Interestingly, LSDV circulating in Bangladesh is 99.99% homologous with two old African field strains—Neethling 2490 and KSGP 0240—which have also been used as attenuated vaccine strains [56,57]. Recent molecular studies on the partial-genome sequencing of the Bangladeshi field strain also suggested a similar genomic pattern [35]. In the phylogenetic tree, there were clear clusters between the LSDV vaccine and the field strains [58]. The full-length sequencing of various LSDV strains collected from recently affected Asian countries showed that the circulating field strains could be divided into two clusters: the so-called recombinant strain cluster [59] and another cluster containing those strains that were more related to the old African LSDV field isolates [60], into which the two Bangladeshi strains isolated in this study were also grouped. This second cluster comprised recent LSDV strains circulating in the Balkans, Caucasus region, and some Asian countries apart from China, Mongolia, Vietnam, Hong Kong, and Taiwan [59,61,62]. The findings of our study confirmed that the genome of the LSDV is stable and that despite the widescale global spread of the virus, genomic changes do not easily occur in natural settings. This agreed with the most recent molecular study by Vandenbussche and coworkers (2022), who stated that the LSDV recombinant strain isolated in the Russian Federation, China, Mongolia, Vietnam, Hong Kong, and Taiwan was a spillover of a poor-quality vaccine. The homologous vaccine was contaminated with several capripox viruses; the study group was able to demonstrate the presence of the recombinant strains in the vaccine vial [63]. Future studies are required to provide further whole-genome sequencing data from different Asian LSDV isolates for a better understanding of the molecular epidemiology of LSD.

## 5. Conclusions

This was the first in-depth study on the clinical pathology of LSD and the complete genome sequence of the Bangladeshi LSDV detected in small holdings of cattle in 2021. Molecular detection methods such as qPCR stood as the most rapid and accurate methods used to confirm LSD infections using various sample types. In typical pathology laboratories, IHC can also be employed to locate the LSDV antigen in skin nodules. Whole-genome sequencing revealed the re-emergence of an older strain of LSDV that currently is circulating in Bangladesh and causing massive outbreaks in different locations of the country. Further investigation through experimental infection with the current field strain may unveil the biological properties of LSDV.

## Data Availability

All required data are available as figures in main text or in the Supplementary Materials. The sequence data were submitted to GenBank and are available under the accession numbers OP688128 and OP688129.

## Supplementary Materials

The following supporting information can be downloaded at: https://www.mdpi.com/article/10.3390/v14112529/s1. Figure S1: Divisional map of Bangladesh (collected from Wikipedia and Google Maps and modified) showing the location of the Mymensingh district where 88 household of five villages with suspected LSD cases were investigated; Figure S2: Box-and-whisker plots describing the clinical manifestations of LSD-affected cattle. (A) Average temperatures recorded (mostly between 38.8 and 40 °C) of the investigated cattle. (B) Range of the age of skin lesions, which varied between 3 and 110 days. (C) Range of the duration of illness in affected cattle, which varied from 1 to 14 weeks; Figure S3: Various skin lesions developed in LSD-affected cattle. (A) Multiple nodules of varying sizes throughout the entire body of an affected cow. (B) Skin laceration in the muzzle of a calf. (C) Severe skin lesions with secondary maggot infection followed by a sloughing off of the top of the skin nodule. (D) Laceration of the lower part of a hind limb. (E) The recovery stage of a skin lesion (scab formation). (F) Swelling of a carpus joint and a single ruptured nodule.
