# Supplementary material for: Clinical Epidemiology, Pathology, and Molecular Investigation of Lumpy Skin Disease Outbreaks in Bangladesh during 2020–2021 Indicate the Re-Emergence of an Old African Strain
Source: Viruses. 2022 Nov 15;14(11):2529. doi: 10.3390/v14112529 (PMC9698944; doi:10.3390/v14112529)
Supplement: Supplementary file 1 [file viruses-14-02529-s001.zip › viruses-1995273-supplementary.pdf]

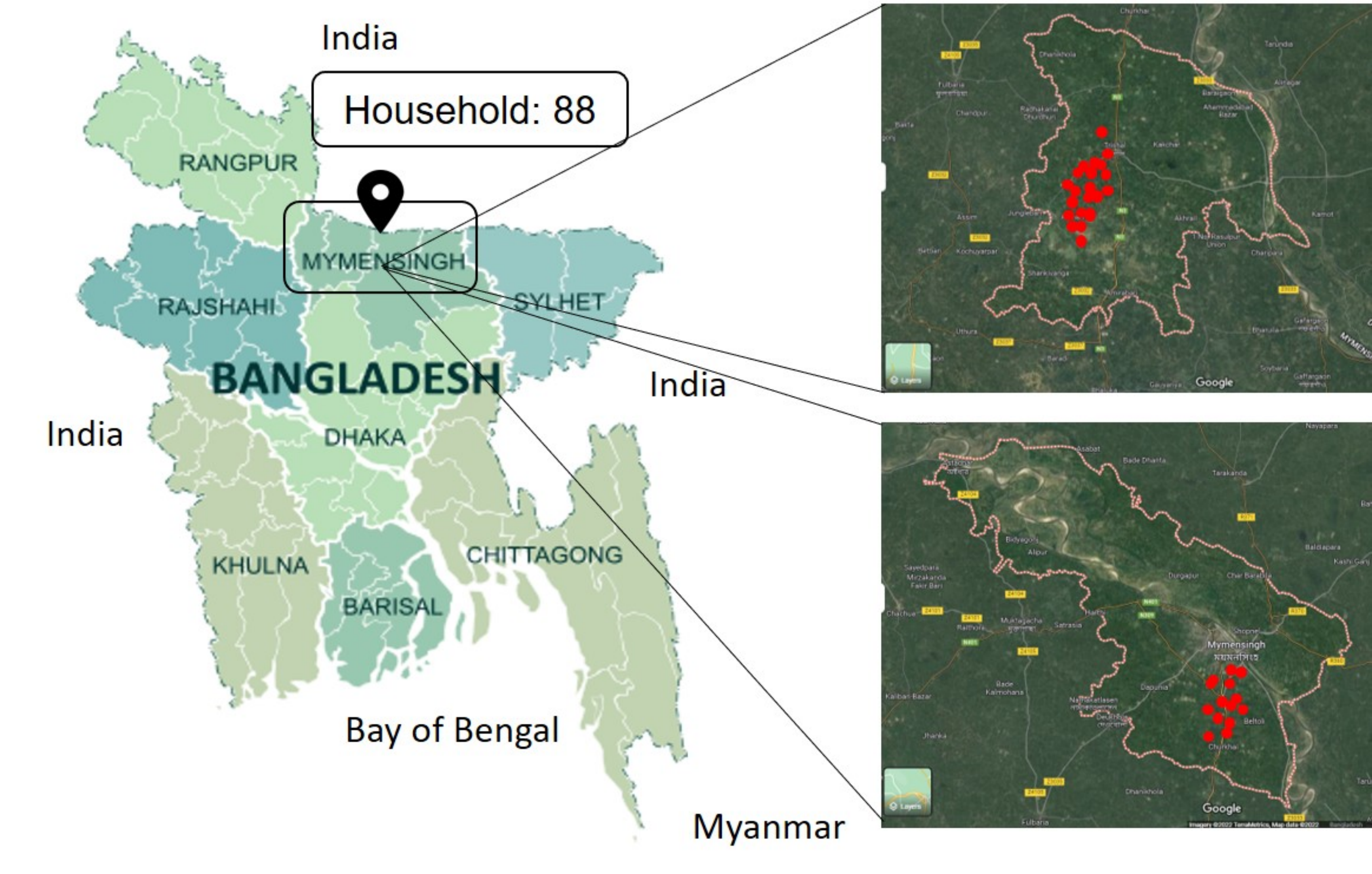
India
Household: 88
RANGPUR
MYMENSINGH
RAJSHAHI
SYLHET
BANGLADESH
India
India
DHAKA
KHULNA
CHITTAGONG
BARISAL
Bay of Bengal
Myanmar
Google
Google

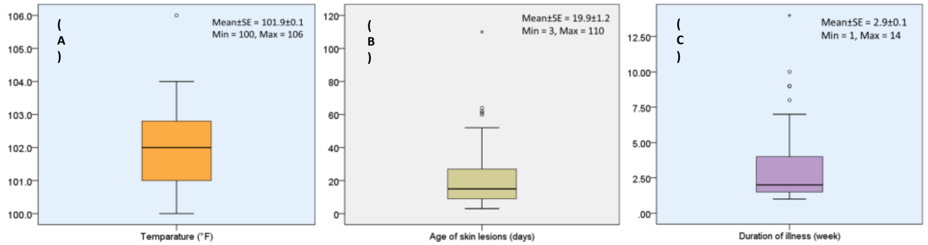

Supplemental Figure S2: Box and whisker plots describes the clinical manifestations of LSD affected cattle. (A) Average temperature recorded mostly in between 102 - 104°F of the investigated cattle (B) Range of the age of skin lesions that varies between 3-110 days. (C) Range of the duration of illness in affected cattle that varies from 1-14 weeks.

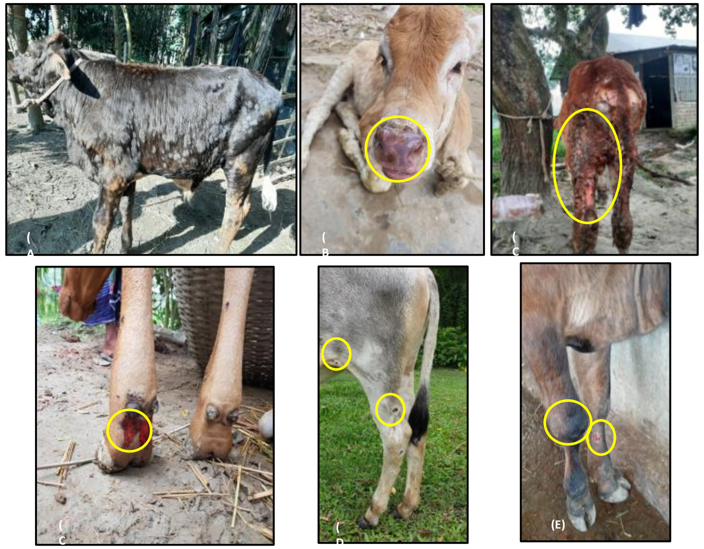

Supplemental Figure S3: Various skin lesions developed in LSD affected cattle (A) Multiple nodules of varying sizes at the entire body part of an affected cattle. (B) skin laceration at the muzzle of a calf. (C) Sever skin lesions with secondary maggot infection followed by sloughing off skin layer. (D) Laceration of the lower part of the hind limb. (E) The recovery stage of skin lesion (scab formation). (F) Swelling of carpus joint and a single ruptured nodule.
